# Risk factors for pressure ulcers from the use of a pelvic positioner in hip surgery: a retrospective observational cohort study in 229 patients

**DOI:** 10.1186/s13037-020-00237-7

**Published:** 2020-04-07

**Authors:** Takuro Ueno, Tamon Kabata, Yoshitomo Kajino, Daisuke Inoue, Takaaki Ohmori, Junya Yoshitani, Ken Ueoka, Yuki Yamamuro, Hiroyuki Tsuchiya

**Affiliations:** Department of Orthopedic Surgery, Kanazawa University Graduate School of Medical Sciences, 13-1 Takaramachi, Kanazawa, Ishikawa 920-8641 Japan

**Keywords:** Hip surgery, Pressure ulcer, Postsurgical care, Lateral decubitus position, Pelvic positioner, Total hip arthroplasty

## Abstract

**Background:**

Intraoperatively acquired pressure ulcers are serious postsurgical complications requiring additional treatment, reoperation, and extended hospitalization. No study has investigated the frequency of the ulcers caused by compression with a pelvic positioner, which is used in hip surgeries to stabilize patients in the lateral decubitus position.

**Methods:**

This retrospective study investigated the risk factors and the frequency of the ulcers caused by the use of pelvic positioners in hip surgeries. The records of patients who underwent surgical procedures under general anesthesia at our institution between January 1, 2016 and March 31, 2018 were reviewed. The inclusion criterion for the assessment of risk factors was hip surgery in the lateral decubitus position stabilized by a pelvic positioner. The exclusion criteria were patients with trauma, missing data, or a pre-existing pressure ulcer. Finally,.the study included 229 patients (265 hip surgeries). All the patients were positioned in the lateral decubitus position with the assistance of either a pelvic positioner, which had a single support fixture located over the pubic symphysis or a double support fixture located over the bilateral anterior superior iliac spine. Intraoperatively acquired pressure ulcers were diagnosed when ulcers were absent on admission and the redness that was observed immediately after surgery remained after 24 h. Multivariate analysis was used to identify factors associated with an increased risk for ulcers.

**Results:**

Ulcers developed in 8 of 1810 (0.44%) patients who underwent orthopedic surgery. Seven of the 265 (2.64%) patients who underwent hip surgery in the lateral decubitus position stabilized by a pelvic positioner developed ulcers. All ulcers were located on areas of the body that were compressed by the pelvic positioner. After identifying controls for patient height (less than 154 cm), surgery duration (longer than 180 min), blood loss (more than 355 ml), and type of pelvic positioner used, we identified the independent risk factors for ulcers to be patient height < 154 cm (adjusted odds ratio, 12.8; *p*-value, 0.032) and the use of pelvic positioners with pubic bone support (adjusted odds ratio, 10.53; p-value, 0.047).

**Conclusion:**

The use of pelvic positioners with pubic bone support should be avoided in patients with a height of < 154 cm to decrease the risk of ulcers.

## Background

Pressure ulcers are localized injuries to the skin and/or underlying tissue caused by pressure with or without shear [1]. Patients undergoing surgery are at a high-risk for developing pressure ulcers, as they must withstand continuous pressure to maintain the necessary position on the operating table. Intraoperatively acquired pressure ulcers are serious postsurgical complications that require additional treatment, reoperation, and extended hospitalization [2]. Their frequency ranges from 2.5 to 66%, [3–7] and their incidence is high in orthopedic surgeries (up to 11.8%) [6]. Other factors associated with ulcers include age, [8–10] sex, [9, 10] low Braden scale score, [9, 11, 12] low body mass index (BMI), [7, 12] diabetes, [13] duration of surgery, [4, 7, 14–16] surgical positioning (prone and park-bench position), [7, 16] application of external force, [7] and the amount of blood loss [7]. In addition, medical device-related pressure ulcers defined as resulting from the use of devices designed and applied for diagnostic or therapeutic purposes [9] have been reported to be increased owing to the development of technology and the increased use of medical devices for patients [17]. The risk of pressure ulcer development in patients with medical devices is reportedly 2.4 times greater than that in other patients [11]. Therefore, ulcers may also be caused by compression with a pelvic positioner, which stabilizes the patient in the lateral decubitus position for hip surgeries including total hip arthroplasty (THA) and pelvic or proximal femoral osteotomy for joint deformities. However, no study has investigated the frequency of ulcers in these particular surgeries nor have they investigated the risk factors specific to hip surgery patients who were stabilized by a pelvic positioner. Therefore, this study investigated the frequency of ulcers caused by a pelvic positioner in hip surgeries and identified the risk factors specifically associated with the use of a pelvic positioner.

## Methods

### Study design

This retrospective study included patients who underwent surgical procedures under general anesthesia at our institution between January 1, 2016 and March 31, 2018. The study data were obtained from the hospital archive system.

### Participants

During the study period, 9087 surgical procedures under general anesthesia were performed at our institution. Of these, 1810 surgical procedures were performed in the orthopedic and spine surgery departments. The inclusion criterion for the assessment of risk factors was hip surgery in the lateral decubitus position stabilized by a pelvic positioner. The pelvic positioner was used in all hip surgeries performed in the lateral decubitus position (*n* = 265) but was not used in any other surgeries performed during the study. The exclusion criteria included patients with trauma, patients with missing data, and patients with a pressure ulcer existing before surgery. We analyzed 265 hip surgeries (229 patients). Of these surgeries, there were 222 primary THAs, 22 THA revisions, 16 pelvic or femoral osteotomies (13 hips for rotational acetabular osteotomy for developmental dysplasia of the hip and 3 hips for femoral osteotomy for deformity correction), and 5 other hip surgeries (1 osteosynthesis for periprosthetic fracture, 1 hardware removal, 1 tumor excision, and 2 irrigation and debridement for infection after THA). All operations were performed single-handedly by a senior surgeon (TK) according to previously described surgical procedures for primary THA [18–23]. Preoperative preparation included the use of a CT-based, 3-D templating and navigation software (CT-based Hip, version 1.0; Stryker Navigation, Freiburg, Germany). In total, 22 THA revisions were performed for frequent dislocation, infection, or aseptic loosening. In 16 surgeries, both acetabular and femoral components were replaced with cementless implants. In 3 of the surgeries, only the acetabular components were replaced and, in an additional three surgeries, only the femoral components were replaced. The acetabular component was inserted with a press-fit fixation using the navigation system. The acetabular component was inserted with press-fit fixation by using the navigation system. Surgeons chose to use either the posterior approach or the anterolateral approach to THA. The posterior approach was used in 21 THAs, and the anterolateral approach was used in 201 THAs. The rotational acetabular osteotomy was performed as described previously [24].

### Position setting

**Fig. 1:**
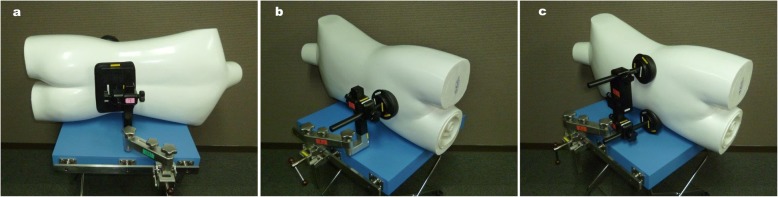
Pelvic positioner (**a**) left, posterior support over the sacrum; (**b**) middle, anterior single support over the pubic symphysis (pubic support type); and (**c**) right, anterior double supports over the bilateral anterior superior iliac spine (ASIS type)

In our hospital, lateral decubitus positioning with the use of a pelvic positioner was performed according to our standard procedures. The patient was placed in the lateral decubitus position on the operating table (Maquet Otesus, Getinge, Japan). The dependent and nondependent upper extremities were placed on armrests with gel pads under the axilla to prevent brachial plexus injury. Both upper extremities were secured with urethane foams (Soft Nurse, Kracie, Japan). The nonoperative lower extremity was secured with urethane foams and bands to prevent peroneal nerve injury. The operative lower extremity was not secured so it could move freely for the surgical procedure. The operative lower extremity was sterilized with an iodine disinfectant from the toes to the ala of the ilium. An intraoperative warming device (Bair Hugger Patient Warming System, 3 M, Maplewood, MN, USA) was used for hypothermia prevention. The pelvic positioner (Flexible lateral positioner; Isomedical Systems, Japan) was used to stabilize the patient in the lateral decubitus position. The compression part of the pelvic positioner was made of a gel pad covered with synthetic material. We used the same posterior support over the sacrum but different anterior support. A single support was used over the pubic symphysis, or double supports were used over the bilateral ASIS (Fig. 1). The patient’s body was stabilized with the type of positioner chosen by the surgeon. The positioning was performed by the surgeon and an assistant (an orthopedic doctor).

### Determination of intraoperatively acquired pressure ulcers

Two nurses and an attending doctor assessed the patient’s skin throughout the perioperative period. In addition, the surgeon assessed their skin immediately after the induction of general anesthesia and at the end of surgery in the operating room. Nurses in the hospital ward assessed the patients’ skin every 24 h after the surgery. The presence of non-blanchable erythema was evaluated using finger pressure. The nurses recorded the results of the skin assessment in the patient record, and pressure ulcers were diagnosed based on the appearance of localized signs that were still observed 24 h after surgery. Intraoperatively acquired pressure ulcers were diagnosed if the nursing records stated that there were no signs of ulcers at admission, redness appeared immediately following surgery, and that the redness remained 24 h after surgery. All pressure ulcers were classified according to the National Pressure Ulcer Advisory Panel staging clinical practice guidelines [1].

### Data collection

All data were obtained from the hospital archive system by the first author (TU). Potential risk factors examined in this study were selected according to previous studies. [5–8, 16] These potential risk factors included age, height, weight, sex, BMI, diabetes, smoking history, steroid use, hemoglobin level, albumin level, length of surgery, blood loss, Braden score, [25] type of pelvic positioner, and surgical procedures. The analyses also included the Braden score recorded at the time of administration as well as 2 days before surgery, and hemoglobin and albumin levels measured 1 day after surgery.

### Statistical analysis

Univariate analyses for each independent variable were performed using the *휒*^2^ test or Fisher’s exact test for categorical variables and the unpaired *t*-test or Mann–Whitney *U* test for continuous variables. For categorical variables, the adjusted standardized residual was calculated to determine which cells contributed the most to the value. Variables with a *p*-value ≤0.05 in the univariate analysis were included in the subsequent multivariate analysis. For continuous variables, the optimal thresholds were determined for the multivariate model using a receiver operating characteristic (ROC) curve at the point showing the highest sum of sensitivity and specificity. Statistical analyses were performed using the SPSS software package (SPSS for Windows, version 23.0; IBM Inc., Armonk, NY, USA). The level of statistical significance was set at a *p*-value < 0.05.

## Results

### Frequency of ulcers

Ulcers were found in 17 patients during the study period. In the department of orthopedic surgery, ulcers developed in 8 patients out of 1810 surgeries (0.44%). Of these 8 patients, 7 developed ulcers after hip surgery performed in the lateral decubitus position with the patient stabilized using the pelvic positioner (2.64%), and one developed in spine surgery performed in the prone position (0.23%) (Table 1). The patient that underwent this spine surgery developed an ulcer on the anterior chest after a thoracic lumbar fusion.

**Table 1:** The frequency of IAPUs in each department

	Operation under GA	Number of IAPUs	Percentage (%)
**All department**	**9087**	**17**	**0.19**
Department of Orthopedic surgery	1810	8	0.44
Department of Neurosurgery	618	6	0.97
Department of Otorhinolaryngology	775	1	0.13
Department of Thoracic and cardiovascular surgery	1377	2	0.15
Other departments	4057	0	
**Department of Orthopedic surgery**	**1810**	**8**	**0.44**
Hip surgery with lateral decubitus position stabilized by a pelvic positioner	265	7	2.64
Spine surgery with prone position	432	1	0.23
Other procedures	1113	0	

*GA* General anesthesia, *IAPU* Intraoperatively acquired pressure ulcer

**Fig. 2:**
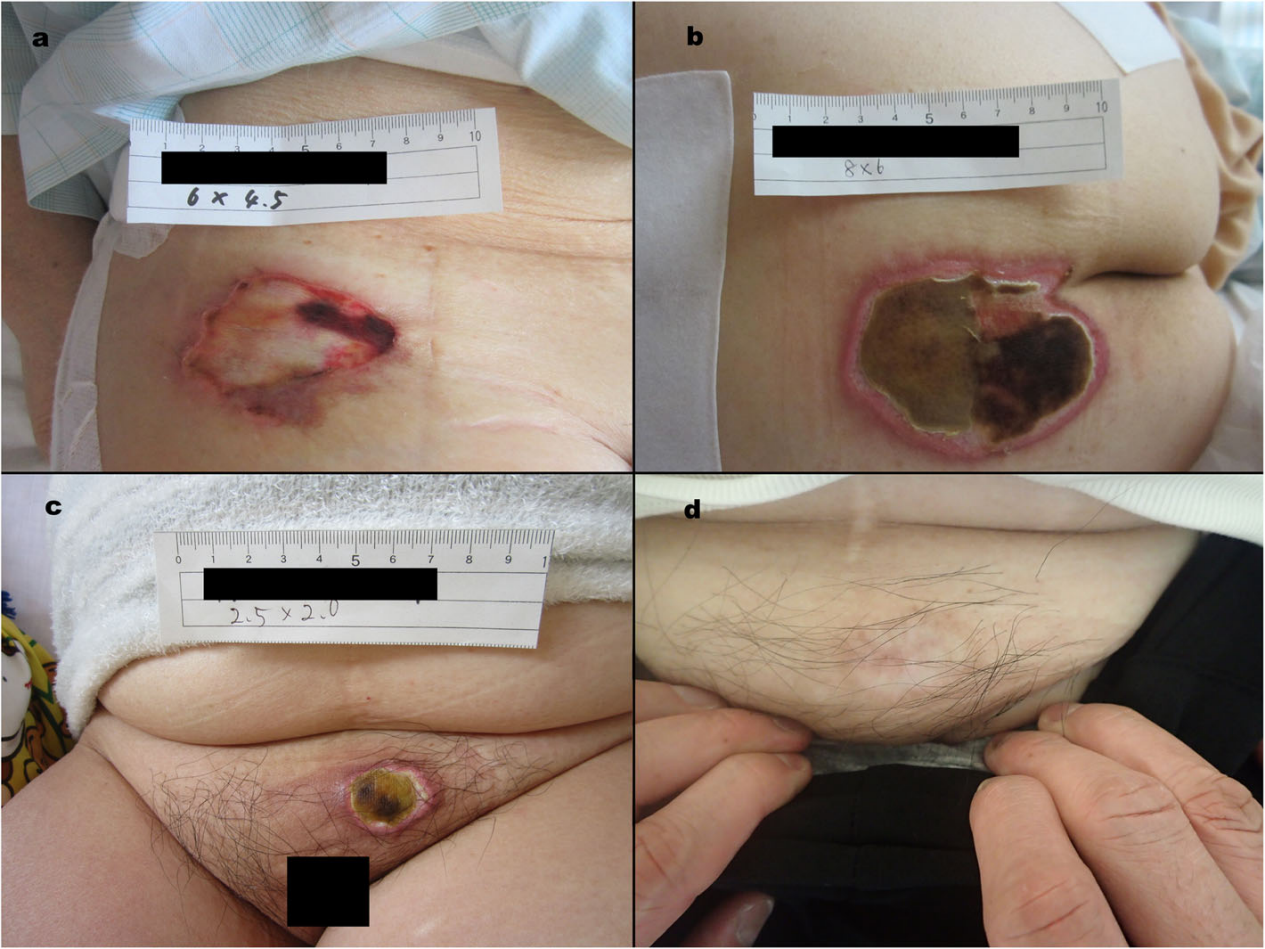
Representative intraoperatively acquired pressure ulcers (**a**) left side of upper column, pressure ulcer developed on the ASIS of patient number 1; (**b**) right side of upper column, PU developed on the sacrum of patient number 2; (**c**) left side of lower column, PU developed on the pubis of patient number 3; and (**d**) right side of lower column, PU in patient number 3 one year after surgery. The numbers of patients who were categorized as having completely healed pressure ulcer are listed in Table 2

The remaining 7 ulcers in hip surgery were caused by the compression of the pelvic positioner for stabilizing the patient in the lateral decubitus position (Table 2). Two patients had a stage II ulcer, and five patients had a stage III ulcer. The pressure ulcers were located on the upper side of the ilium in 1 patient where an ASIS support was used, on the sacrum of 1 patient where a pubic support type was used, and on the pubic symphysis of 5 patients where a pubic support type was used (Fig. 2). All pressure injuries were managed with hydrocolloid dressings and were followed by an expert team consisting of a dermatologist and nurses. All pressure ulcers were assessed as healed at the final follow-up.

**Table 2:** Baseline data of patients with IAPUs developed in hip surgeries by compression of the pelvic positioner for stabilizing the lateral decubitus position

Patient	Age	Sex	HT (cm)	WT (Kg)	BMI (Kg/m^2^)	Braden Scale	Hb (g/dl)	Alb (g/dl)	Procedure	Condition	Length of procedure (mins)	Amount of bleeding (ml)	Pelvic positioner types	Affected region	Size (cm^2^)	NPUAP stage
1	92	F	140	50	25.3	20	13.3	4.2	THA	OA	226	360	ASIS	upside of ilium	6.0*4.5	II
2	61	F	154	52	21.9	23	13.1	4.4	THA	OA	153	200	Pubis	sacrum	8.0*6.0	III
3	70	F	142	64	31.6	22	12.5	4.4	THA	OA	246	460	Pubis	pubis	2.5*2.0	III
4	76	F	145	59	28.3	22	13.9	4.2	re-THA	aseptic loosening	515	420	Pubis	pubis	3.5*2.0	III
5	12	M	150	41	18.2	23	13.6	4.9	Osteotomy	DDH	326	745	Pubis	pubis	2.5*1.2	II
6	41	F	151	48	21.1	23	12.3	4.3	Osteotomy	DDH	225	460	Pubis	pubis	3.0*1.5	III
7	74	M	168	59	20.9	22	12.3	4.1	re-THA	frequent dislocation	181	240	Pubis	pubis	4.5*3.0	III

*HT* Height, *WT* Weight, *BMI* Body mass index, *Hb* Hemoglobin, *Alb* Albumin, *NPUAP* National Pressure Ulcer Advisory Panel, *F* Female, *M* Male, *THA* Total hip arthroplasty, *OA* Osteoarthritis, *ASIS* Anterior superior iliac spine, *DDH* Developmental dysplasia of the hip

Further evaluation was performed for patients undergoing hip surgeries to determine the risk factors. The mean ± standard deviation (SD) height was 150 ± 9.4 cm in patients with ulcers, and 156.8 ± 9.6 cm in patients without ulcers (*p* = 0.039). The mean ± SD length of surgery was 267.4 ± 122.0 min in patients with ulcers, and 172.5 ± 66.1 min in patients without ulcers; (*p* = 0.0026). The mean ± SD amount of bleeding was 412.1 ± 179.3 ml in patients with ulcers, and 298.2 ± 242.5 ml in patients without ulcers (*p* = 0.041). No significant differences were observed in other continuous variables. No differences were noted between patients with or without ulcers in the categorical variables of sex, steroid use, diabetes, and smoking history. However, the pubic support type of pelvic positioner was used more frequently in patients who acquired ulcers (85.7%) than those who did not (32.6%) (*p* = 0.0034) (Table 3). In addition, THA revision and pelvic or proximal femoral osteotomy were performed more frequently in patients who acquired ulcers than those who did not (*p* = 0.011, adjusted standardized residual in revision THA = 1.97, adjusted standardized residual in pelvic or proximal femoral osteotomy = 2.53). The optimal thresholds were determined for categorical variables with a *p*-value of less than 0.05 in univariable analysis. The thresholds were less than 154.1 cm for patient height, longer than 180.5 min for length of surgery, and more than 355 ml for blood loss (Table 4).

**Table 3:** Data comparison between patients with IAPUs and those without IAPUs

	With IAPUs (*n* = 7)	Without IAPUs (*n* = 258)	*p* value
Age (years)	60.9 ± 26.6	60.2 ± 14.3	0.49
HT (cm)	150.0 ± 9.4	156.8 ± 9.6	0.04
WT (Kg)	53.2 ± 7.9	56.9 ± 12.3	0.53
BMI (Kg/m^2^)	23.9 ± 4.7	23.1 ± 4.2	0.77
Hemoglobin (g/dl)	13.0 ± 0.7	13.0 ± 1.5	0.97
Albumin (g/dl)	4.4 ± 0.3	4.2 ± 0.4	0.33
Length of surgery (minutes)	267.4 ± 122.0	172.5 ± 66.1	0.0026
Amount of bleeding (ml)	412.1 ± 179.3	298.2 ± 242.5	0.041
Braden Scale score	22.1 ± 1.1	21.2 ± 0.6	0.22
Sex (Male:Female)	2:5	62:196	0.78
Steroid use (Yes:No)	0:7	14:244	0.53
Diabetes (Yes:No)	1:6	33:225	0.91
Smoking (Yes:No)	0:7	35:223	0.30
Pelvic positioner (PS:ASIS)	6:1	84:174	0.0034
Surgical procedure (THA:reTHA:PO:Ir)	3:2:2:0	219:20:14:5	0.011

The data are shown as mean ± SD for continuous variables or number for categorical variables

Residual analysis was performed in surgical procedures; revision THA and pelvic or proximal femoral osteotomy were more frequently performed in in patients with IAPUs than in those without (*p* = 0.011, adjusted standardized residual in revision THA = 1.97, adjusted standardized residual in pelvic or proximal femoral osteotomy = 2.53)

*IAPU* Intraoperatively acquired pressure ulcer, *HT* Height, *WT* Weight, *BMI* Body mass index, *NPUAP* National Pressure Ulcer Advisory Panel, *THA* Total hip arthroplasty, *re THA* Revision THA, *PO* Periacetabular osteotomy, *PS* Pubic symphysis type, *ASIS* Anterior superior iliac spine type, *DDH* Developmental dysplasia of the hip

**Table 4:** ROC analysis to determine the thresholds

Variables	AUC	Thresholds	Sensitivity	Specificity
HT (cm)	0.728	154.1	0.857	0.585
Length of surgery (minutes)	0.834	180.50	0.857	0.760
Amount of bleeding (ml)	0.726	355	0.714	0.767

*AUC* Area under the curve, *HT* Height

Multivariate analysis including patient height, length of surgery, amount of bleeding, pelvic positioner types, and surgical procedures confirmed that patient height < 154.1 cm (adjusted odds ratio, 12.75; *p*-value, 0.032) and the use of a pelvic positioner with a pubic support (adjusted odds ratio, 10.53; *p*-value, 0.047) were independent risk factors associated with ulcers (Table 5).

**Table 5:** Multivariate analysis

		95%CI		
	Adjusted odds ratio	lower	upper	*p* value
Height < 154 (cm)	12.75	1.25	129.90	0.032
Length of surgery > 180 (minutes)	13.12	0.70	245.29	0.085
Amount of bleeding > 355 (ml)	0.86	0.09	8.43	0.900
Pubic type pelvic positioner	10.53	1.03	107.73	0.047
Revision THA or Osteotomy	1.68	0.25	11.39	0.600

Cox-Snell R^2^ = 0.087; Nagelkerke R^2^ = 0.40

*CI* Confidence interval

## Discussion

To the best of our knowledge, this study is the first to evaluate the frequency of ulcers caused by compression from the use of a pelvic positioner and the associated risk factors. Ulcers were found in 7 of 265 hip surgeries (2.64%). All ulcers developed on areas of the patients’ bodies that had contact with the pelvic positioner: 1 patient on the upper side of the ilium, 1 patient on the sacrum, and 5 patients on the pubis. Although surgeries from various departments were analyzed together, the frequency of ulcers ranged from 2.5 to 66% in previous studies [3–7].

In a prospective study by Ling et al. [7]., ulcers were reported in 48 cases out of 1940 patients (2.5%) in neurosurgery, orthopedic surgery, pediatric surgery, and cardiovascular surgery which is similar to this study. However, in their study,27% (13 patients of 48 patients) of patients with ulcers underwent cardiac surgery, which requires long-term bedrest.

In contrast, more than 1 day of bedrest after surgery was not required for any patients involved in this study. This suggests that patients in this study were more active in the early postoperative period compared to those in the other study. Conversely, Versluysen [6] reported that 66 out of 100 patients (66%) with femoral fractures developed ulcers. That reported frequency is significantly higher than that of this study. This difference may be due to the differences in patient demographics and movement ability. The patients in that study were older and had limited movement ability before surgery compared to those in this study, who primarily had degenerative diseases.

The frequency of ulcers varied greatly due to differences in surgical procedure/position, the area of the body where ulcers developed, and patient demographics. Therefore, the frequency and risk factors for ulcers specific to the lateral decubitus position stabilized by a pelvic positioner are unclear. In addition, we experienced several ulcers that were caused by a pelvic positioner in clinical practice that motivated us to conduct this study.

Multivariate analysis confirmed that shorter patient height (< 154.1 cm) and the use of a pubis support type pelvic positioner were independent risk factors associated with ulcers. No difference was observed in other potential risk factors including age, sex, Braden score, BMI, diabetes, blood albumin level, and smoking history between patients with ulcers and those without. This suggests that the traditionally reported factors associated with pressure ulcers have no strong predictive value for ulcers caused by a pelvic positioner. In addition, previous studies report that the Braden scale is not completely applicable to surgical patients because it is used to evaluate pressure ulcers in older and immobile patients [7].

The application of exertion of external forces, such as pressure hemostasis, and utilization of a restraint strap were reported as risk factors for ulcers [7]. Pelvic positioners, which are devices that apply external forces, are widely used in hip surgeries to stabilize the patient in the lateral decubitus position throughout surgery [26–28]. This study found a higher frequency of ulcers in surgeries that used pelvic positioners compared to those that did not (Table 1). It is reasonable for surgeons to prefer to firmly stabilize the patient’s pelvis with a pelvic positioner; however, this may put great pressure on the contact area between the patient and the positioner (pubis, ASIS, or sacrum), especially in THA, because a large degree of pelvic movement during hip arthroplasty leads to great variability in pelvic orientation at implantation. This causes wide variability in the final orientation of the acetabular component and can increase risk of acetabular component malalignment [26, 27, 29]. Additionally, if the pressure from the pelvic positioner exceeds the capillary interface pressure (23–32 mmHg), [30] it will cause direct capillary blood occlusion and tissue ischemia, which may lead to pressure ulcers. This study also attempted to determine which types of pelvic positioners were more likely to cause ulcers. To that end, we used 2 types of positioners (pubic support and ASIS). Although both types are widely used to stabilize the patient in the lateral decubitus position, the choice of positioning device depends on the institution’s policies or surgeon’s preference. In our institution, surgeon preference dictated the type of pelvic positioner used. Although no clear criteria for the use of a pelvic positioner have been established, the pubic-support type was preferred for use in acetabular osteotomies. In addition, the pubic support-type was used for a few THAs. The frequency of ulcers caused by the pubic support type of positioner was 6.7% (6 out of 90 cases). Of these 6 patients, 5 ulcers developed in the pubic region, and 1 ulcer developed in the sacral region. According to a previous study, both the pubic and sacral regions are common locations for developing pressure ulcers [4]. These results demonstrate the prevalence of ulcers in anatomic locations that are in contact with direct pressure. On the other hand, only 1 ulcer among 175 patients (0.57%) developed with the use of an ASIS type of pelvic positioner. The ulcer developed on the ASIS of the operative side. Therefore, our study showed the highest frequency of ulcers with the used of the pubic support type of pelvic positioner (6.7%) compared to, not only the frequency with the use of another type (ASIS type) of pelvic positioner (0.57%) but also that of the orthopedic surgery department (0.44%), other departments (neurosurgery, 0.97%; otorhinolaryngology, 0.13%; thoracic and cardiovascular surgery, 0.15%), or in the entire department (0.19%). As no other studies have evaluated the types of pelvic positioners in terms of risk for ulcers, the mechanism for the extremely high frequency of pressure ulcers associated with the application of the pubic support type is unclear. However, pelvic movement during THA is reported to be greater in patients stabilized by the pubic support type of pelvic positioners than in those stabilized by the ASIS type [26]. This indicates that the compression of the pubic region resulted in an unstable fixation compared to the compression of the bilateral ASIS. This difference leads to a different pressure distribution or friction resulting in high frequency of pressure ulcers.

This hypothesis is unusual because the study was conducted retrospectively so further analysis is warranted to measure the direct pressure on the patient’s body from the pelvic positioner and, thereby, to confirm this hypothesis.

This study showed a higher frequency of ulcers during THA revision and osteotomy in univariable analysis. However, after controlling for other factors, no differences were found between the procedures. Similarly, intraoperative blood loss was higher in patients who developed ulcers in the univariable analysis, and no differences were found in the multivariable analysis. This finding may be attributed to the surgery duration, which was a confounding variable between the occurrence of ulcers and two other variables (surgical procedures or amount of blood loss).

The duration of surgery is a well-known risk factor for ulcers. [4, 7, 14–16 A study conducted across multiple departments reported that the mean length of surgery for patients who developed ulcers was 234 min [4].

Even though surgical procedures included in this study were limited to hip surgeries performed in the lateral decubitus position with the patient stabilized by a pelvic positioner, the mean length of surgery that resulted in ulcers (267 min) and that which did not result in ulcers (172 min) were similar to those in other studies. Furthermore, ROC analysis revealed that the optimal threshold for the length of surgery was > 181 min. Although it did not reach a statistically significant level, after controlling for other factors, the risk of ulcers increased 13 times when the length of surgery was more than 181 min (*p* = 0.085).

Low BMI has been reported to be a risk factor for ulcers [7, 12] because thinner patients have additional bony prominences. However, this study confirmed that short body height was an independent factor associated with an increased risk of pelvic positioner-related ulcers, whereas BMI and body weight were not. In addition, this study indicated that the risk of ulcers increased 12.8 times when the patient’s height was < 154 cm (*p* = 0.032). To the best of our knowledge, no other study has described the relationship between body height and ulcers. Therefore, our study indicates that a specific relationship may exist between short body height (instead of BMI or weight) and the development of ulcers specific to the lateral decubitus position stabilized by a pelvic positioner. We suspected that, in shorter patients, the relatively oversized pad of the pelvic positioner might abnormally distribute the pressure and, in turn, lead to ulcers. However, further investigation with the addition of pressure monitoring is needed to clarify this result.

Our analysis indicates that the pubic support type of pelvic positioner should be avoided for patients of short height and also for prolonged surgeries. If the pubis support type is used, additional interventions should be introduced to protect the patient’s skin. Therefore, in our institution, we discontinued the use of the pubic support type of pelvic positioner in April 2018. Further study is needed to verify the ulcer actually decreased afterwards.

This study has several limitations. First, the sample size of ulcers was small. This was a problem when the multivariable logistic analysis was considered. However, to identify specific risk factors, we only analyzed the patients who had hip surgery in the lateral decubitus position and were stabilized with a pelvic positioner in a multivariable model. This helped to improve the accuracy to fit with the multivariable model. Second, the patient’s body was manually compressed by the pelvic positioner and the compression strength was not quantified. Therefore, the compression strength may vary between patients. In addition, all surgeries were performed in the same department in the same institution by the same surgeon; therefore, although this consistency minimized any confounding factors related to patient positioning, the results are not generalizable. Further study involving multiple surgeons are needed to determine if the risk is specific to this surgeon, position devices or the method of application. Third, no clear criteria were set in terms of how types of pelvic positioners were selected. Multivariable analysis confirmed that the use of the pubic support type of pelvic positioner increased the risk for ulcers regardless of the surgical procedure or patient height. Finally, because this was a retrospective study, further prospective studies with large samples are needed.

## Conclusion

The frequency of ulcers was high in hip surgeries performed in the lateral decubitus position with the patient stabilized by a pelvic positioner (7 ulcers of 265 surgeries, 2.64%). In addition, we discovered an association between an increased risk of developing ulcers and shorter height when using a pubic support type of pelvic positioner and prolonged surgery. We suspect that increased pressure on the area of the patient’s body that came into contact with the pubic support type of pelvic positioner contributed to the high frequency. However, further prospective studies are warranted to investigate the relationship between pressure and patient height or pelvic positioner types.

## Data Availability

The datasets used and analyzed during the current study are available from the corresponding author on reasonable request.

## Abbreviations

Alb: Albumin
ASIS: Anterior superior iliac spine
AUC: Area under the curve
BMI: Body mass index
CI: Confidence interval
DDH: Developmental dysplasia of the hip
F: Female
GA: General anesthesia
HT: Height
Hb: Hemoglobin
ICU: Intensive care unit
IAPUs: Intraoperatively acquired pressure ulcers
M: Male
NPUAP: National Pressure Ulcer Advisory Panel
OA: Osteoarthritis
PU: Pressure ulcer
ROC: Receiver operating characteristic
SD: Standard deviation
THA: Total hip arthroplasty
WT: Weight
